# Creatinine, but not cystatin C, varies diurnally in critically ill children: a retrospective analysis from a tertiary pediatric intensive care unit

**DOI:** 10.1007/s00431-026-06996-2

**Published:** 2026-04-24

**Authors:** Robert Frithiof, Mats B. Eriksson, Jonathan Cedernaes, Ali Reza Modiri, David Smekal, Anders Larsson

**Affiliations:** 1 Department of Surgical Sciences, Anaesthesiology and Intensive Care Medicine, Uppsala University, Uppsala University Hospital, SE-751 85 Uppsala, Sweden; 2 NOVA Medical School, New University of Lisbon, 1099-085 Lisbon, Portugal; 3 Department of Medical Sciences, Clinical Chemistry and Pharmacology, Uppsala University, Uppsala University Hospital, 751 85 Uppsala, Sweden

**Keywords:** Critical care, Creatinine, Cystatin C, Diurnal, Glomerular filtration rate: Pediatric

## Abstract

Accurate evaluation of kidney function is vital in the pediatric intensive care unit (PICU), where even small changes in cystatin C and creatinine concentrations can affect clinical decision-making. Diurnal patterns in renal biomarkers have been reported in adults, but their relevance in critically ill children remains unclear. Understanding whether sampling time contributes to biological variability is essential for reliable interpretation of kidney function tests. This retrospective study included 8619 cystatin C and 9314 creatinine results collected in a tertiary PICU between April 2014 and September 2025. The hourly distribution of sampling and hourly biomarker percentiles (0.10, 0.25, and 0.50) were evaluated across the 24-h cycle. Diurnal variation was quantified using coefficients of variation (CVs). Sampling was strongly clustered in the early morning, with 38.6% of cystatin C and 37.4% of creatinine samples drawn at 5:00–5:59 AM. The 0.10–0.50 percentiles of cystatin C showed minimal diurnal variation (CV 4.5–6.3%). Creatinine exhibited slightly greater variability, with CVs of 7.4–11.6% across the same percentiles. Median creatinine was significantly higher in the afternoon/evening than in the early morning, while cystatin C showed no clinically relevant hourly fluctuations.

*Conclusion*: In critically ill children, cystatin C demonstrates limited diurnal variation, while creatinine shows modest but measurable fluctuations across the 24-h period. These findings suggest that cystatin C is relatively robust to sampling time in the PICU, whereas creatinine may vary enough to influence interpretation in borderline cases. Incorporating knowledge of sampling time may improve the accuracy of kidney function assessment and AKI classification in pediatric critical care.

**Table:** 

**What is Known:** •* Creatinine and cystatin C can show biological and circadian variability in adults and older children, with creatinine generally exhibiting greater within-day fluctuation than cystatin C.*	
**What is New:** • *In critically ill children, cystatin C shows minimal diurnal variation, whereas creatinine displays modest but measurable time-of-day–related increases, indicating that sampling time may influence creatinine interpretation but not cystatin C in the PICU setting.*

**What is Known:**

•* Creatinine and cystatin C can show biological and circadian variability in adults and older children, with creatinine generally exhibiting greater within-day fluctuation than cystatin C.*

**What is New:**

• *In critically ill children, cystatin C shows minimal diurnal variation, whereas creatinine displays modest but measurable time-of-day–related increases, indicating that sampling time may influence creatinine interpretation but not cystatin C in the PICU setting.*

## Introduction

Accurate assessment of kidney function is essential in the pediatric intensive care unit (PICU), where critically ill children are at heightened risk of acute kidney injury (AKI) and associated poor outcomes due to hemodynamic instability, systemic inflammation, nephrotoxic medications, and multiorgan dysfunction [1]. Creatinine and cystatin C remain the most widely used endogenous biomarkers for estimating glomerular filtration rate (GFR) in children, yet both exhibit substantial biological and analytical variability. In critically ill pediatric populations, interpreting small changes in these biomarkers is particularly challenging, as even modest shifts may influence AKI staging, therapeutic decisions, and prognosis [2, 3]. Biological variation over 24 h has been demonstrated for creatinine and cystatin C, with cystatin C exhibiting some diurnal rhythmic variation and creatinine showing greater within-subject variability in individuals without CKD; reference change values (RCVs) for all eGFR equations were within 13–20% in both study groups [4, 5].

Although small increases in serum creatinine can be clinically relevant and form the basis of the KDIGO definition of stage 1 AKI (0.3 mg/dL within 48 h), several pediatric studies indicate that stage 1 AKI often carries a substantially lower prognostic impact than higher AKI stages [6]. Moreover, analytical and biological variation in creatinine measurement can approach the magnitude of such small changes, suggesting that fluctuations within this range may not reflect clinically meaningful kidney injury [7].

Creatinine, although well established, is influenced by muscle mass, hydration status, tubular secretion, and the effect of certain medications. In children, rapid developmental changes and heterogeneous underlying diagnoses further complicate interpretation. Cystatin C has emerged as a potentially more sensitive biomarker because it is less dependent on muscle mass and may detect early declines in GFR. However, cystatin C can also be affected by inflammation, corticosteroid therapy, thyroid dysfunction, and assay-related factors, conditions frequently encountered in the PICU. Understanding the natural variability of these biomarkers is therefore crucial for avoiding misclassification of kidney function [8, 9].

Growing evidence in adults and older children suggests that kidney biomarkers, including creatinine and cystatin C, may follow circadian patterns linked to endogenous rhythms in renal blood flow, filtration, and tubular handling [4, 5, 10]. Even small diurnal fluctuations might impact clinical interpretation when biomarker changes are used to diagnose AKI or guide fluid and medication management. However, whether such diurnal variation exists, persists, or is altered in critically ill pediatric patients remains unknown. PICU patients are exposed to continuous interventions, such as mechanical ventilation, vasoactive medications, sedatives, altered sleep–wake cycles, and irregular feeding patterns, that may disrupt or override circadian physiology. Consequently, diurnal variation may be blunted, exaggerated, or highly variable in this population. In a prospective longitudinal cohort study, practice patterns for deciding when to administer “as needed” analgesic and sedation medications differed between day and night shifts [11].

Clarifying whether critically ill children exhibit clinically relevant diurnal variations in creatinine and cystatin C, similar to adults [5, 10, 12], in the PICU is essential for determining whether the timing of sample collection should be considered when evaluating renal function. If biomarker levels fluctuate predictably across the 24-h cycle, sample timing could influence AKI classification or mask early trends. Conversely, if concentrations remain stable, clinicians can interpret changes with greater confidence regardless of sampling time.

The aim of this study is to investigate whether cystatin C and creatinine exhibit diurnal variation in critically ill children admitted to a tertiary PICU.

By evaluating real-world longitudinal biomarker data, we seek to determine whether sample timing contributes meaningfully to biological variability and whether it should be incorporated into clinical interpretation and future guidelines for pediatric kidney function assessment.

## Materials and methods

### Samples

Routine requests from the pediatric intensive care unit at Uppsala Pediatric Hospital sent to the Departments of Clinical Chemistry and Pharmacology at Uppsala University Hospital (Uppsala, Sweden) were included in the dataset. Blood was drawn into Li-heparin gel tubes (LH PSTII 366567, BD Vacutainer Systems, Plymouth, UK). The study covered the period from April 15th, 2014, to September 30th, 2025, yielding a total of 8619 cystatin C and 9314 creatinine results. The laboratory reports cystatin C as a concentration in mg/L and estimated glomerular filtration rate (eGFR) in mL/min/1.73 m^2^ and creatinine in µmol/L (mg/dL = µmol/L × 0.011312) and eGFR in mL/min/1.73 m^2^. Data extraction was performed in accordance with the approved ethical protocol, and only limited demographic and analytical variables were retrieved: patient age (in years), sex, sampling time in hours, and the corresponding cystatin C and creatinine concentrations. No personal identifiers were collected. Ethical approval was granted by the Regional Ethical Review Board in Uppsala, Sweden (Dnr 01–367). Reporting of this retrospective, observational study followed the STROBE (Strengthening the Reporting of Observational Studies in Epidemiology) guidelines [13].

### Instruments

During the initial years of the study, creatinine and cystatin C were measured on the Architect ci8200 platform (Abbott Laboratories, Abbott Park, IL, USA) using creatinine reagents from Abbott Laboratories and cystatin C reagents from Gentian (Moss, Norway). In February 2021, the assay was migrated to the Cobas Pro c503 system (Roche Diagnostics, Rotkreuz, Switzerland), employing reagent kits from Roche Diagnostics. The method transition was evaluated by parallel analysis of patient samples on both instruments to ensure analytical comparability. All creatinine testing was performed with IDMS calibrated enzymatic methods. For these assays, imprecision was evaluated using two quality control levels, designated level 1 and level 2 for practical purposes. These levels typically yield slightly different CVs, although no specific levels are mandated other than that they be clinically relevant (Table 1).

**Table 1:** Imprecisions of cystatin C and creatinine, respectively, are assessed using two clinically relevant quality control levels, which may yield slightly different CVs

Analyte	Instrument	Level 1	Level 1 (CV)	Level 2	Level 2 (CV)	Year
Creatinine	Cobas	80	0.02	360	0.02	2024
Creatinine	Cobas	80	0.02	360	0.02	2023
Creatinine	Abbott Architect	70	0.03	350	0.02	2019
Creatinine	Abbott Architect	70	0.03	350	0.02	2020
Cystatin C	Cobas	1.1	0.02	4.3	0.03	2024
Cystatin C	Cobas	0.95	0.03	4.1	0.03	2023
Cystatin C	Abbott Architect	0.7	0.04	3.3	0.04	2019
Cystatin C	Abbott Architect	0.7	0.04	3.3	0.04	2020

### Statistical calculations

Differences in plasma creatinine and cystatin C concentrations between two predefined time periods (before and after 13:00H, respectively) were assessed using the Mann–Whitney *U* test. The null hypothesis assumed no temporal difference in concentrations. Data is expressed as median and IQR. A *p* < 0.05 was considered significant. Statistical processing and data visualization were carried out using Excel 365 (Microsoft Corp., Seattle, WA, USA) and Statistica version 10 (Tibco Software, Palo Alto, CA, USA).

## Results

### Basic patient information

A total of 8619 cystatin C (3815 girls and 4804 boys) and 9314 creatinine (4154 girls and 5160 boys) results were reported during the study period.

The median age of the girls assayed for cystatin C was 3 years (IQR 1–8 years), and the median cystatin C value was 0.90 mg/L (0.69–1.29 mg/L). The median age of the boys sampled for cystatin C was also 3 years (IQR 1–7 years), and the median cystatin C value was 0.89 mg/L (0.70–1.22 mg/L).

The median age of the girls assayed for creatinine was 3 years (IQR 1–8 years), and the median creatinine value was 26.5 µmol/L (19.7–40.9 µmol/L). The median age of the boys sampled for creatinine was also 3 years (IQR 1–8 years), and the median creatinine value was 26.5 µmol/L (20.3–39.2 µmol/L).

### Diurnal variation of cystatin C and creatinine across study years

For the years 2014–2020, the 0.5-percentile mean of cystatin C was derived from values obtained during hours 0–13 (0.98; *n* = 3698) and 14–23 (0.93; *n* = 340), respectively.

For the period 2021–2025, the mean of the 0.5 percentile of cystatin C was estimated from measurements collected during hours 0–13 (0.85; *n* = 3980) and 14–23 (0.85; *n* = 447), respectively.

During 2014–2020, the 0.5-percentile mean of creatinine was obtained from samples drawn in hours 0–13 (26.5; *n* = 4022) and 14–23 (31.4; *n* = 408), respectively.

For the years 2021–2025, the mean value corresponding to the 0.5 percentile of creatinine was calculated from results recorded during hours 0–13 (26.2; *n* = 4205) and 14–23 (33.3; *n* = 570), respectively.

### Hourly distribution of cystatin C sampling

**Fig. 1:**
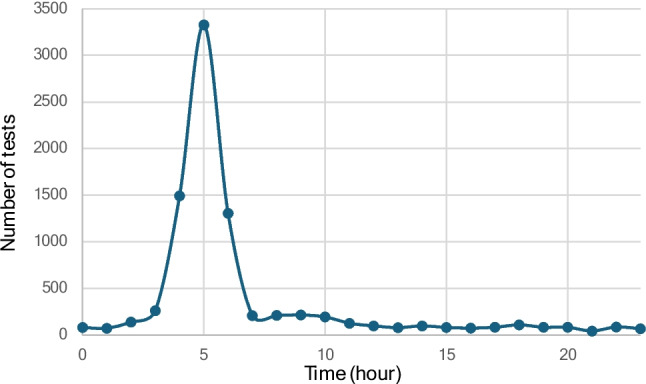
Number of cystatin C requests for each hour of the 24-h period

The highest number of cystatin C samples was collected at 5:00–5:59 AM, accounting for 3327 samples (38.6%). Substantial numbers were also drawn at 4:00–4:59 AM (1492 samples) and 6:00–6:59 AM (1305 samples). During the remaining hours of the 24-h period, approximately 100 samples per hour were collected (Fig. 1).

### Hourly variation in cystatin C concentrations

**Fig. 2:**
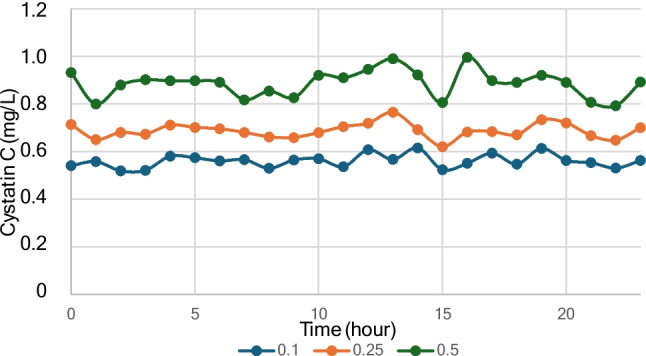
Hourly sampling of cystatin C throughout the day, illustrating the 0.1, 0.25, and 0.5-percentile levels

Cystatin C concentrations were evaluated across hourly percentiles (0.10, 0.25, and 0.50 [median]) for the full 24-h period. The 0.10, 0.25, and 0.50 percentiles exhibited minimal diurnal variation. The coefficients of variation (CVs) across the 24-h cycle were 4.9% for the 0.10 percentile, 4.5% for the 0.25 percentile, and 6.3% for the median (Fig. 2).

### Hourly distribution of creatinine sampling

**Fig. 3:**
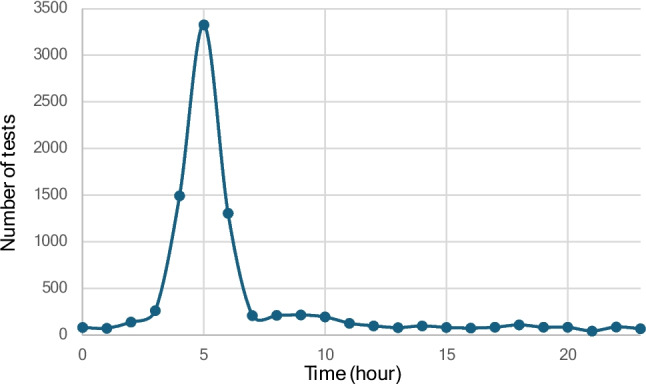
Number of creatinine requests for each hour of the 24-h period

The highest number of creatinine samples was collected at 5:00–5:59 AM, accounting for 3483 samples (37.4%). Substantial numbers were also drawn at 4:00–4:59 AM (1556 samples) and 6:00–6:59 AM (1386 samples). During the remaining hours of the 24-h period, approximately 100 samples per hour were collected (Fig. 3).

### Hourly variation in creatinine concentrations

**Fig. 4:**
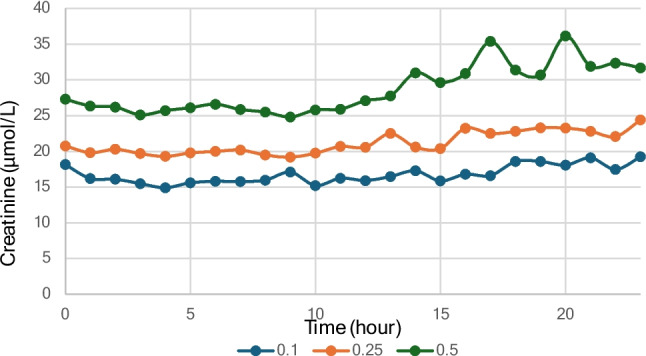
Hourly sampling of creatinine throughout the day, illustrating the 0.1, 0.25, and 0.5-percentile levels

Creatinine concentrations were evaluated across hourly percentiles (0.10, 0.25, and 0.50 [median]) for the full 24-h period. The 0.10, 0.25, and 0.50 percentiles exhibited slightly higher diurnal variation than cystatin C. The coefficients of variation (CVs) across the 24-h cycle were 7.6% for the 0.10 percentile, 7.4% for the 0.25 percentile, and 11.6% for the median. The median PM creatinine values were higher than the median AM values. During the morning, the mean of the 50th percentile of plasma creatinine concentrations was 26.0 µmol/L (CV: 2.7), increasing in the afternoon to 31.7 µmol/L (CV: 7.4), corresponding to a 22% increase (Fig. 4).

With 13:00 arbitrarily defined as the cutoff time point, plasma creatinine concentrations were significantly elevated after this time at the 0.1, 0.25, and 0.5 percentiles, whereas no corresponding increase was observed for cystatin C (Table 2).

**Table 2:** Results from Mann–Whitney *U* statistics testing the hypothesis of a difference in plasma creatinine and plasma cystatin C concentrations when sampled before compared to after 13:00, stratified based on 0.1, 0.25, and 0.5 concentration percentiles

	*Z*	*p* value
Creatinine 0.1	− 3.19	0.001
Creatinine 0.25	− 3.82	< 0.001
Creatinine 0.5	− 4.11	< 0.001
Cystatin C 0.1	− 0.52	n.s
Cystatin C 0.25	− 0.12	n.s
Cystatin C 0.5	− 0.09	n.s

## Discussion

In this large retrospective study of more than 17,000 biomarker measurements from a tertiary pediatric intensive care unit, we evaluated whether cystatin C and creatinine exhibit clinically meaningful diurnal variation. Understanding the natural within-day variability of kidney biomarkers is important in the PICU, where even small changes can alter AKI staging, prompt clinical intervention, or influence the interpretation of trends in renal function. Our findings provide new insight into the stability of these commonly used markers under real-world critical care conditions.

A central finding of this study is the remarkable stability of cystatin C across the 24-h cycle. The lowest three percentiles (0.10, 0.25, and 0.50) demonstrated coefficients of variation below 6.5%, with no consistent peak–trough pattern suggestive of circadian influence. This suggests that cystatin C is minimally affected by diurnal physiology in critically ill children, aligning with reports from adult studies in stable outpatients [5]. Importantly, the PICU population is subjected to sedation, mechanical ventilation, vasopressors, and irregular sleep–wake patterns, factors that might be expected to dampen or distort circadian rhythms. The stability of cystatin C in this setting reinforces its robustness as an endogenous marker of GFR, even when surrounding physiological cycles are disrupted.

In contrast, creatinine showed modest diurnal variation, with CVs ranging from 7.4 to 11.6% and higher median values in the afternoon and evening. This is consistent with findings in adults, where creatinine displays reproducible diurnal patterns that may relate to circadian changes in renal blood flow, tubular secretion, or metabolic activity [14]. In the pediatric ICU, additional factors such as intermittent feeding, fluctuating muscle perfusion, and medication schedules may contribute to these patterns. While the observed variation in creatinine is modest, it may have practical implications: Differences of this magnitude could influence AKI staging in borderline cases, especially when creatinine changes are near diagnostic thresholds.

The strong clustering of sampling at 4–6 AM reflects standard nursing workflow and laboratory routines rather than clinical need. Importantly, the heavy concentration of early-morning samples does not appear to bias cystatin C interpretation although an interaction with the circadian rhythm of cortisol has been conjectured to explain higher cystatin C levels in the morning in healthy adults [5]. For creatinine, a high proportion of samples collected in the early morning may reduce the likelihood of capturing peak afternoon values.

Clinicians should be aware that early-morning samples do not necessarily reflect the physiologically increased creatinine values that may occur in the afternoon. The increase in the mean of the 50th percentile of plasma creatinine observed in the afternoon compared with the morning corresponded to a 22% rise. The correlation between creatinine concentrations and eGFR is non-linear [15, 16], which means that the difference in eGFR could be higher than 22%. In a clinical study, an increase of 15.6% in plasma creatinine was considered clinically meaningful [17].

One potential concern is whether small fluctuations in creatinine or cystatin C might reflect true kidney injury. However, there is no evidence that AKI exhibits diurnal variation, and if such variation was present, both biomarkers would be expected to change in parallel, since the vast majority of samplings were performed at the same time. This supports the interpretation that the small, time of day–related changes observed here do not represent clinically meaningful kidney injury.

Patient median and lower percentile values provide a stable and outlier-resistant baseline for biomarker monitoring. It enhances trend detection, reduces noise, and supports more precise interpretation than relying solely on maximum–minimum values, population reference intervals, or single measurements [18]. Biomarkers are often not normally distributed but instead typically follow a log-normal distribution, largely because disease processes such as AKI lead to elevated levels that disproportionately affect the upper percentiles. As a result, one approach to deriving reference intervals from laboratory databases is to focus only on the central or lower part of the distribution [19].

Our findings have several implications for clinical practice. First, cystatin C appears sufficiently stable to be interpreted without adjustment for sampling time, supporting its reliability in the PICU environment. Second, creatinine interpretation may benefit from awareness of diurnal patterns, particularly when changes are small or when AKI criteria hinge on narrow thresholds. Third, for research studies and epidemiological analyses, accounting for sampling time may reduce noise and improve the precision of creatinine-based estimates of kidney function.

This study has limitations. It is a single-center study. The retrospective design precluded assessment of clinical covariates such as illness severity, fluid balance, medication exposure, or feeding patterns, all of which may influence biomarker concentrations. We also lacked repeated samples from individual patients at multiple time points, limiting evaluation of within-patient trajectories. However, the large sample size, extended study period, and diverse PICU population strengthen the generalizability of the findings.

In conclusion, cystatin C shows minimal diurnal variation in critically ill children, whereas creatinine demonstrates modest fluctuations across the 24-h cycle. These findings support the use of cystatin C as a stable marker of kidney function and highlight the importance of sample timing when interpreting creatinine in the PICU. Future studies incorporating matched clinical data and repeated within-patient sampling may further clarify the determinants and clinical consequences of diurnal variability in pediatric kidney biomarkers.

## Data Availability

Data are available from the corresponding author upon reasonable request.
